# High genetic diversity in a small population: the case of Chilean blue whales

**DOI:** 10.1002/ece3.998

**Published:** 2014-03-20

**Authors:** Juan P Torres-Florez, Rodrigo Hucke-Gaete, Howard Rosenbaum, Christian C Figueroa

**Affiliations:** 1 Instituto de Ciencias Ambientales y Evolutivas, Universidad Austral de ChileIndependencia 641, Valdivia, Chile; 2 Centro Ballena Azul/Blue whale CenterIndependencia 641, Valdivia, Chile; 3 Instituto de Ciencias Marinas y Limnológicas, Universidad Austral de ChileIndependencia 641, Valdivia, Chile; 4 Ocean Giants Program, Wildlife Conservation Society2300 Southern Boulevard, Bronx, New York, 10460, USA; 5 Sackler Institute for Comparative Genomics, American Museum of Natural HistoryCentral Park West at 79th Street, New York, New York, 10024-5192, USA; 6 Instituto de Ciencias Biológicas, Universidad de Talca2 Norte 685, Talca, Chile

**Keywords:** *Balaenoptera musculus*, Chile, conservation genetics, low population size, microsatellites loci, mitochondrial DNA

## Abstract

It is generally assumed that species with low population sizes have lower genetic diversities than larger populations and vice versa. However, this would not be the case for long-lived species with long generation times, and which populations have declined due to anthropogenic effects, such as the blue whale (*Balaenoptera musculus*). This species was intensively decimated globally to near extinction during the 20th century. Along the Chilean coast, it is estimated that at least 4288 blue whales were hunted from an apparently pre-exploitation population size (k) of a maximum of 6200 individuals (Southeastern Pacific). Thus, here, we describe the mtDNA (control region) and nDNA (microsatellites) diversities of the Chilean blue whale aggregation site in order to verify the expectation of low genetic diversity in small populations. We then compare our findings with other blue whale aggregations in the Southern Hemisphere. Interestingly, although the estimated population size is small compared with the pre-whaling era, there is still considerable genetic diversity, even after the population crash, both in mitochondrial (*N* = 46) and nuclear (*N* = 52) markers (H_d_ = 0.890 and H_o_ = 0.692, respectively). Our results suggest that this diversity could be a consequence of the long generation times and the relatively short period of time elapsed since the end of whaling, which has been observed in other heavily-exploited whale populations. The genetic variability of blue whales on their southern Chile feeding grounds was similar to that found in other Southern Hemisphere blue whale feeding grounds. Our phylogenetic analysis of mtDNA haplotypes does not show extensive differentiation of populations among Southern Hemisphere blue whale feeding grounds. The present study suggests that although levels of genetic diversity are frequently used as estimators of population health, these parameters depend on the biology of the species and should be taken into account in a monitoring framework study to obtain a more complete picture of the conservation status of a population.

## Introduction

There is an understanding that the quantity and quality of genetic diversity of populations may influence their viability (Frankham 2010). However, such effects may be constrained in extremely small populations, whose probability of persistence has been severely threatened by natural or anthropogenic factors, thus highlighting the question about the importance of genetic diversity in conservation biology (e.g., Caro and Laurenson 1994). It is sometimes predicted that reductions in population sizes negatively impact the genetic diversity of populations and therefore limit the capacity of species to face threats such as diseases and global climate change, among others (Frankham 2005). This loss of genetic diversity is a result of increased genetic drift in small populations (Gilpin and Soulé 1986; Soulé 1987). Because genetic drift acts more rapidly in small populations, overall genetic diversity is expected to be roughly proportional to the size of a population and this indeed appears to be the case as Frankham (1996) reviewed taking into account 23 different studies. In addition to increased genetic drift, the frequency of consanguineous mating may also increase and further reduce levels of diversity (Westemeier et al. 1998; Madsen et al. 1999), which may trigger a significant reduction in population fitness (Frankham 2005).

Two main features of genetic diversity can be emphasized when studying the fitness of natural populations. Firstly, the loss of genetic diversity can proceed through mechanisms such as inbreeding depression, genetic drift, or higher genetic load of populations (Charlesworth and Charlesworth 1987; Charpentier et al. 2005; Grueber et al. 2008; Bouzat 2010; Hedrick and Fredrickson 2010). Secondly, the lower evolutionary potential of decimated populations is expected to show a restricted ability to respond to stochastic environmental changes (Bouzat 2010).

Severe demographic reductions and population bottlenecks due to anthropogenic causes (habitat fragmentation, over-exploitation) are well documented, which can cause loss of genetic variability, higher levels of inbreeding, and reductions in individual fitness (Hedrick and Miller 1992; Lande 1994; Mills and Smouse 1994; Lynch et al. 1995; Frankham et al. 1999; Hoelzel 1999; Weber et al. 2004); however, this relationship is not always linear. Thus, not all large populations will exhibit high genetic diversities (Amos and Harwood 1998), and not all decimated populations will show a reduced genetic diversity (e.g., Gonzáles-Suárez et al. 2010; Hoffman et al. 2011). A number of studies propose that the impact of population decline on genetic variation depends considerably on life-history traits that affect population growth (Kuo and Janzen, 2004; Hailer et al., 2006; Lippé et al., 2006). Processes driving the loss of genetic diversity may be buffered by intrinsic biologic traits, specifically long generation times that can result in small populations that appear genetically diverse despite periods of substantial decline.

**Figure 1:**
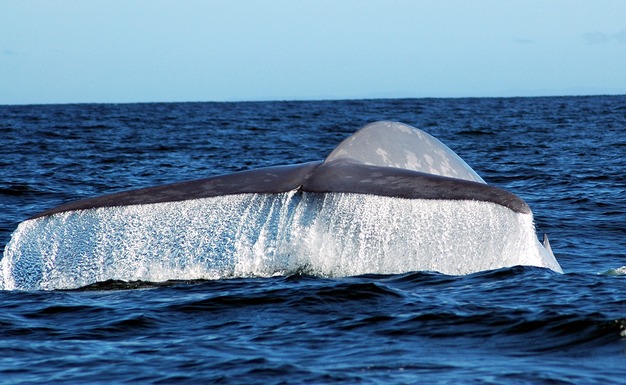
Blue whale in Corcovado Gulf waters.

One of the most emblematic examples of the negative impacts of human activities on marine biodiversity is the significant decrease in whale populations (Roman and Palumbi 2003). Although population decreases for this group have been relatively well quantified and documented, its impact on the genetic diversity of populations is poorly understood (Palumbi and Roman 2007; Jackson et al. 2008), especially for those species driven to near extinction, like the blue whale (*Balaenoptera musculus*; Fig. 1). Blue whales became the principal target for the whaling industry throughout the world during the early 1900s (Clapham et al. 1999). Only in the Antarctic, ca. 330,000–360,000 blue whales were killed during the 20th century (Tønnessen and Johnsen 1982), not including the illegal whaling after its catch was banned by International Whaling Commission (IWC) in 1966. Despite the cease of whaling, blue whale populations have not increased enough to reach pre-exploitation sizes, still remaining between 10,000 and 25,000 individuals globally (3–11% of the estimated population size at the year 1911) (Branch et al. 2004; Vié et al. 2009; Williams et al. 2011). According to the International Union for Conservation of Nature (IUCN) Red List of Threatened Species, the current conservation status of the blue whale is “*Endangered*” (En) (Vié et al. 2009). While three subspecies are recognized, two of them inhabit the Southern Hemisphere: “true” or “Antarctic” blue whale (*B.m.intermedia*) and “pygmy” blue whale (*B.m.brevicauda*), both remaining among the most endangered baleen whales (Clapham et al. 1999). Although blue whales found northern the 60º S have been proposed to belong the “pygmy” subspecies, reports about the distribution limits, breeding areas, feeding grounds, whaling effects on population dynamics, and genetic diversity remain very limited (Reilly and Thayer 1990; Hucke-Gaete et al. 2004; Branch et al. 2007a,b; LeDuc et al. 2007; Buchan et al. 2010; Attard et al. 2012; Sremba et al. 2012). Migration patterns of the species are not well understood, being proposed that blue whales belonging to the “true” morphotype can perform long migrations between high-latitude feeding grounds to low low-latitudes where they breed, while the “pygmy” morph may migrate between mid-latitude feeding grounds to low-latitude to breed (Branch et al. 2007a). However, the migration routes, extent of migrations, and the number of individuals performing migrations are poorly known.

Recently, a feeding ground consisting of 232 individual blue whales (coefficient of variation CV = 0.68) was discovered off the coast of southern Chile (Corcovado Gulf) (Hucke-Gaete et al. 2004, 2010). This area corresponds to one of the most important feeding aggregation areas for blue whales in the Southern Hemisphere (i.e., feeding hotspot) and is characterized by the presence of mother-calf pairs as well as solitary individuals during the austral summer and early fall season (Hucke-Gaete et al. 2004; Galletti Vernazzani et al. 2012). Presumably, the 60 years of blue whale harvesting in Chile (1908–1967) account for the small size of this feeding hotspot (Williams et al. 2011). Indeed, at least 4288 blue whales were harvested along the Chilean coast, explaining why the number of blue whales in Chile (based on 1997–1998 estimates) is still one order of magnitude less than during the prewhaling (Williams et al. 2011). After almost five decades since the cessation of whaling, the population still remains at a low population size. One possible hypothesis for this lack of recovery to pre-exploitation numbers is the loss of genetic diversity due to genetic drift and/or other phenomenon associated with severe reductions in population numbers resulting from the combination of small population size and the apparently isolated situation.

In this study, we aim to determine whether a drastic reduction in individuals during the 20th century has influenced the genetic diversity (mitochondrial and microsatellite polymorphisms) as well as the current effective population size of Corcovado Gulf blue whales. In addition, we test whether this reduction in individuals shows any signs of a genetic bottleneck and discusses our results in the context of blue whale life-history traits and small population genetic diversity consequences. As there are no direct antecedents of the genetic diversity in the original population before the intense exploitation of blue whales, we are assuming a hypothetical panmictic population large enough to closely match an idealized population. This work presents data that can be valuable for assessing future conservation priorities of blue whales, such as stocks or management units (Palumbi and Cipriano 1998), and for establishing the role that this mid-latitude feeding ground plays during the seasonal migration of Southeastern Pacific blue whales.

## Methods

### Sample collection

**Figure 2:**
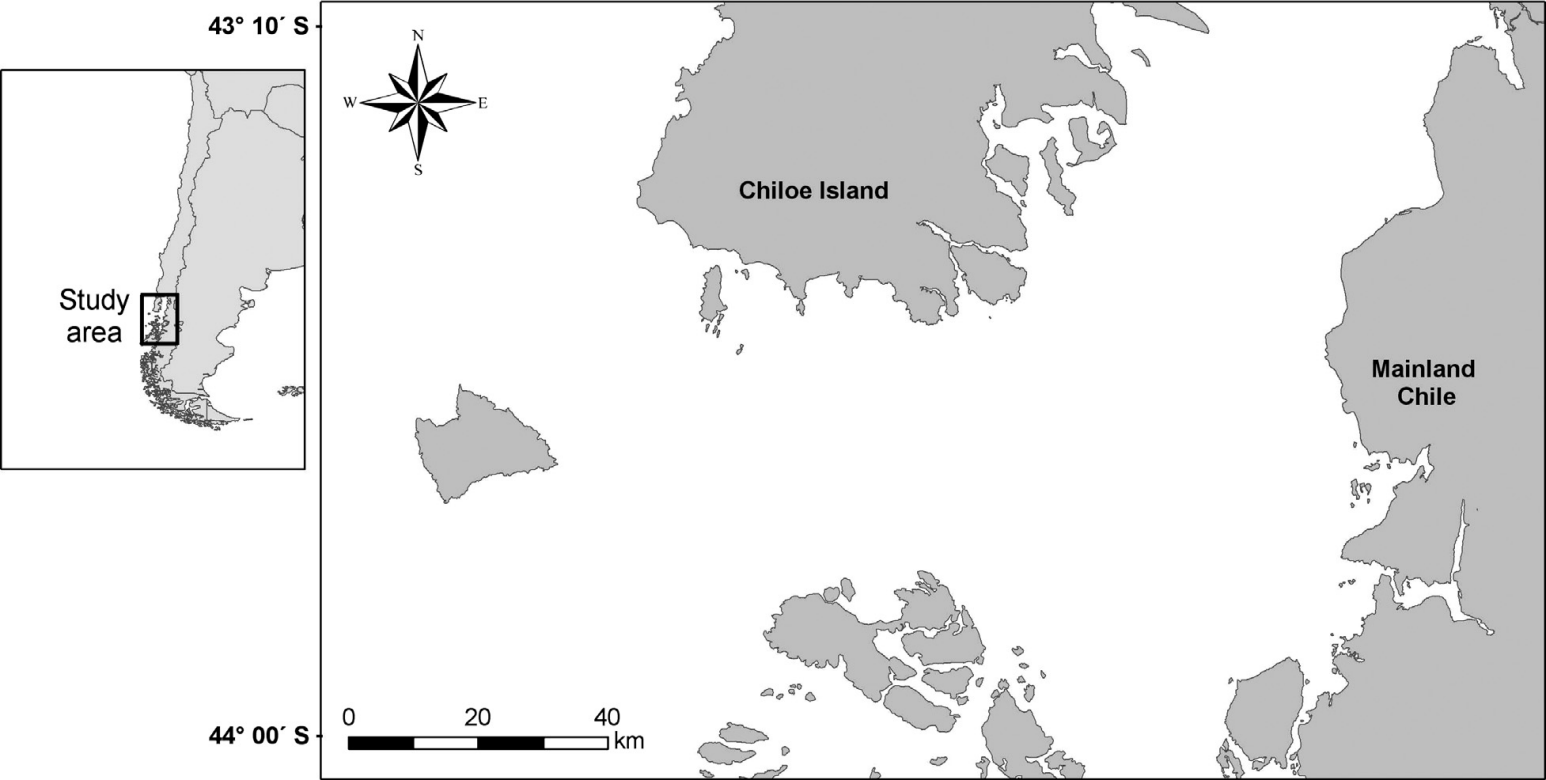
Corcovado Gulf area where samples were collected.

A total of 59 blue whale tissue samples were obtained from the Corcovado Gulf area, located at the northern Chilean Patagonia. Skin samples were collected by biopsy dart procedures using a crossbow or modified rifle (Lambertsen 1987; Krützen et al. 2002), during the blue whale feeding seasons over seven consecutive summers (January to April, 2004–2010) (Fig. 2). Samples were kept in 20% dimethyl sulfoxide (DMSO) saturated with sodium chloride (Amos and Hoelzel 1991) or 95% ethanol, and stored at −20°C until processed.

### Genetic methods

Genomic DNA was extracted using the DNeasy® Blood and Tissue Kit (Qiagen, Chatsworth, CA, USA). To characterize the neutral polymorphism in this blue whale feeding ground, seven microsatellite loci previously described were used using PCR conditions as described previously (Buchanan et al. 1996; Valsecchi and Amos 1996; Palsbøll et al. 1997; Bérubé et al. 2000). Microsatellite genotyping was performed following LeDuc et al. (2007) using an ABI-PRISM 310 automated sequencer (Perkin Elmer). Alleles and genotypes for each microsatellite locus in each sample were identified using GeneMapper® 4.0 software (Thermo Fisher Scientific Inc., Waltham, MA) and recorded in an Excel sheet. The genotype of each sample was recorded as the combination of all alleles amplified for all loci (multilocus genotype).

In addition, a fragment of 421 bp of the mitochondrial DNA control region was amplified by PCR (Primers DLP 1.5 and DLP 5; Baker et al. 1996) and sequenced in both directions using a 3730xl DNA Analyzer (Thermo Fisher Scientific Inc.). Sequences were edited visually with SEQUENCHER 3.0 (Gene Codes Corporation, Ann Arbor, MI, USA) and aligned with CLUSTALX 2.0 (Larkin et al. 2007).

### Microsatellite data analysis

Possible scoring errors (e.g., stutter bands, allele dropout, and null alleles) were checked with MICROCHECKER v.2.2.3 (Van Oosterhout et al. 2004), and 10% of the samples were regenotyped to confirm correct calling of alleles. No scoring errors were detected. To identify matching samples, genotype identities were tested based on a multilocus genotype in GENALEX 6.4 (Peakall and Smouse 2006). The probability of identity P_(ID)_ (i.e., two unrelated individuals from the same panmictic population having an identical genotype by chance) was estimated using two different algorithms included in the same software: the Hardy–Weinberg [HW] P_(ID)_ and the more conservative measure Sib P_(ID)_ (Taberlet and Luikart 1999; Waits et al. 2001). Thus, the probability of identity using seven microsatellite loci was 7.7 × 10^−8^ for unrelated individuals and 1.9 × 10^−3^ for full-siblings, indicating that the microsatellite loci chosen were indeed suitable for accurately discriminating among individual genotypes. Deviations from Hardy–Weinberg equilibrium (HWE) at each locus, heterozygote deficiency, and linkage disequilibrium (LD) for each pair of loci were performed in GENEPOP v.4.0 (Rousset 2008). Deviations tests from HWE and LD were conducted using the program settings for Markov chain parameters (10,000 dememorization steps, 1000 batches, 10,000 iterations per batch) and corrected for simultaneous comparisons with the sequential Bonferroni test (Rice 1989). Genetic diversity was measured as the number of alleles per locus (N_a_), the number of effective alleles per locus (N_e_), and the mean number of alleles per locus (A: allelic diversity). Observed (H_O_) and expected (H_E_) heterozygosities, and the inbreeding coefficient *F*_is_ were all computed using EXCEL MICRO-SATELLITE TOOLKIT 3.1 (Park 2001).

As effective population size estimates as well as bottleneck tests can be biased by a subpopulation structuring (Chikhi et al. 2010), we explored whether whales coming from different breeding groups in the Corcovado Gulf belong to a same population using a Bayesian clustering analysis in STRUCTURE v2.3.1 (Pritchard et al. 2000). The analyses were run considering a range of *k* = 1–4 clusters for four iterations with 100,000 repetitions after a burn-in period of 100,000. The Δk method was used to select the most likely value of k (Evanno et al. 2005).

### Effective population size estimates and bottleneck tests

In order to estimate historical (prewhaling era) and contemporary (postwhaling era) effective population sizes, two different approaches were used. Historical effective population size: the coalescent theory was used to jointly estimate mutation-scaled effective population size as Θ = x*μ*Ne, where *x* is 4 for nuclear data of diploid organisms and N_e_ is the historical effective population size, performed in MIGRATE (Beerli and Felsenstein 2001). Three replicates were run using a Brownian motion mutation model with constant mutation rates (5 × 10^−4^) and starting parameters based on *F*_ST_ calculations. A uniform prior distribution was used to estimate Θ (ranging from 0 to 10). Following a burn-in of 50,000 iterations, each run visited a total of 1,000,000 parameter values and recorded 20,000 genealogies at a sampling increment of 50. A static heating scheme was used at four temperatures (1, 1.5, 3, and 6) to efficiently search the genealogy space. Contemporary effective population size: an approximate Bayesian computation (ABC) analysis was used as implemented in the software OneSamp (Tallmon et al. 2008), running 50,000 iterations with a minimum (N_e_) of 50 and a maximum of 1,500 individuals. Two prior estimates of Θ (2–12) based on previous population size estimates were used (Williams et al. 2011).

To evaluate a recent population decrease, evidence of a genetic bottleneck within the Corcovado Gulf was explored using the analytical method described in Cornuet and Luikart (1996) and implemented in BOTTLENECK v. 1.2.02 (Piry et al. 1999). This analysis is based on the assumption that during population reductions (i.e., bottlenecks), rare alleles are lost quickly through genetic drift because only a few individuals in the population may carry them (Hedrick 2005); however, rare alleles contribute relatively little to the expected heterozygosity under a mutation-genetic drift equilibrium. Thus, in bottlenecked populations, expected heterozygosity calculated from the observed number of alleles is lower than the actual observed heterozygosity (Cornuet and Luikart 1996). Excess in observed heterozygosity can be used to identify populations that have suffered a genetic bottleneck. For this analysis, a two-phase mutation model (TPM) (95% single-step mutations; 5% multistep mutations; 1000 iterations) was used as recommended by Piry et al. (1999). To obtain the probability values for heterozygosity excess, a one-tailed Wilcoxon signed-rank test was performed (due to the number of loci and small sample size).

A second analysis to infer a population bottleneck is based on the *M*-ratio, *k*/*r*, where *k* is the total number of alleles and *r* is the overall range in allele size (Garza and Williamson 2001). In reduced populations, alleles are lost via genetic drift, but this loss of alleles affects *k* and *r* differently: *k* is reduced with each lost allele, whereas only the loss of the largest or the smallest allele reduces *r*. Therefore, recently reduced populations are expected to have lower *M*-ratios than populations at equilibrium (Garza and Williamson 2001). The M-ratio was calculated using the softwares M_P_VAL and CRITICAL_M, determining the value of the observed *M-ratio* and the critical *M-ratio* (Mc) (Garza and Williamson 2001). The parameter setting was as follows: Δg = 3.5 (average size of multistep mutations) as recommended by Garza and Williamson (2001), *ps *= 0.95 (proportion of one- step mutations), and Θ = 3 and Θ = 1 (being Θ = 4Ne*μ*, where *Ne* is the effective population size and *μ* is the mutation rate). Although no linear relationship exists between the population size N and the effective population size Ne (Palstra and Fraser 2012), two likely values for Θ were considered assuming a pre-exploitation Ne of 1500 and 500 blue whales, based on Williams et al. (2011), and a typical mutation rate of *μ *= 5 × 10^−4^ for mammalian microsatellites. The significance of the resulting *M*-ratios was determined by comparison against a distribution of all possible *M* values calculated from 10,000 theoretical populations in mutation-drift equilibrium. Using conventional criteria, a significant reduction in population size was inferred if fewer than 5% of the replicates fall below the observed value of *M*.

### Mitochondrial DNA data analysis

The mtDNA sequences obtained in this study were matched to previously reported haplotypes (Leduc et al. 2007; Attard et al. 2010; Sremba et al. 2012) using COLLAPSE v1.2 (available from http://darwin.uvigo.es) and DnaSP v5.0 (Rozas et al. 2003). Haplotype diversity (Hd) (Nei 1987), mean number of pairwise differences between sequences (k) (Kimura 1980; Tajima 1983), and nucleotide diversity (*π*) (Nei 1987) were calculated using ARLEQUIN v3.5.1.2 (Excoffier and Lischer 2010). In order to establish the neutrality of the marker and to explore for possible population expansions, Tajima's *D* test, Fu's *F* statistics (1000 replicates), R2 test (1000 replicates), and a mismatch distribution test were conducted in DnaSP (Tajima 1989; Fu 1997). A median joining network (95% of confidence level) (Bandelt et al. 1999) was constructed using NETWORK 4.6 (available from http://www.fluxus-engineering.com/sharenet.htm) to investigate the relationships among blue whale mtDNA haplotypes found in the Corcovado Gulf.

### Genetic diversity comparisons with other populations and phylogenetic reconstruction

Mitochondrial DNA nucleotide and haplotype diversities in the Corcovado Gulf feeding ground were compared to those reported for other blue whale feeding grounds: Antarctic Ocean (LeDuc et al. 2007; Sremba et al. 2012) and Australia (Perth Canyon and Bonney upwelling) (Attard et al. 2010). For that purpose, sequences were trimmed to 360 bp. A gross genetic diversity (GGD) (Nº haplotypes/Nº of samples) was calculated for comparison purposes between different areas. A comparative analysis among areas was not possible using microsatellites due to the availability of published data and because different loci have been used in other blue whales studies.

To evaluate ancestral relationship among mtDNA haplotyes found in the four areas (Corcovado Gulf, Antarctic Ocean and both Australian feeding grounds), a phylogenetic tree was constructed using a maximum likelihood algorithm implemented in TREEFINDER (Jobb et al. 2004). The evolutionary model HKY + G (Hasegawa–Kishino–Yano with gamma distribution) was selected in the same software using the AICc (Akaike information criterion corrected). Support for the groupings was estimated with 1000 bootstrap replications. Sequences were obtained from GenBank under the accession numbers HQ130726 to HQ130731; EU093921 to EU093962; and JN801048 to JN801070 (LeDuc et al. 2007; Attard et al. 2010; Sremba et al. 2012).

## Results

A total of seven samples were identified as duplicates based on multilocus microsatellite genotype and mtDNA haplotype matching analyses. Therefore, the duplicated samples were not included for further analyses. Similarly, samples showing more than one missing data type for microsatellite genotypes or gaps into the mtDNA sequences were discarded. Hence, the final data set consisted of 52 samples genotyped at six or more microsatellite loci and 46 samples sequenced for the mtDNA control region, all samples coming from the Corcovado Gulf.

### Microsatellite diversity

The number of different alleles per locus ranged between three and 12 with a mean allele number of 7.71 (SE 1.15) per locus. No locus showed a significant deviation from HWE after sequential Bonferroni correction, and no global heterozygote deficiency was detected (Table 1). Similarly, no significant evidence of linkage disequilibrium between pairs of loci was observed. Observed heterozygosities ranged from 0.365 to 0.846 for all loci. The global observed heterozygosity (0.692) was lower than the expected (0.738; *P *>* *0.05) (Table 1).

**Table 1:** Genetic diversity in the Corcovado Gulf area detected using seven microsatellite loci.

	N	Na	Ne	A	Ho	He	Fis	Reference
GATA417	52	12	6.090	11.695	0.846	0.836	−0.003	Palsbøll et al. 1997;
GT23	45	7	4.535	7.000	0.689	0.780	0.127^*^	Bérubé et al. 2000;
Gata098	52	8	4.060	7.964	0.769	0.754	−0.011^*^	Palsbøll et al. 1997;
Gata028	52	9	4.595	8.932	0.731	0.782	0.076	Palsbøll et al. 1997;
DlrFCB17t	52	3	1.684	3.000	0.365	0.406	0.110	Buchanan et al. 1996;
EV37t	50	5	3.757	4.991	0.640	0.734	0.138	Valsecchi and Amos 1996;
ACCC392	52	10	5.516	9.529	0.804	0.819	0.028	Palsbøll et al. 1997
GLOBAL		7.714 (1.149)	4.320 (0.535)	7.587 (1.099)	0.692 (0.060)	0.730 (0.056)	0.062 (0.023)	

N, Number of individuals analyzed for each locus; Na, Number of alleles for each locus; Ne, Number of effective alleles for each locus; A, Allelic diversity; Ho, Observed heterozygosity; He, Expected heterozygosity; Fis, Inbreeding coefficient. Standard Error between brackets, ^*^significance.

The Bayesian clustering analysis did not show any evidence of admixture in the Corcovado Gulf. Although the Δ*k* method of Evanno et al. (2005) supported *k* = 2, bar plot showing individual assignment probabilities for the given *K* was close to 0.5, which is an indicative of actual *K* = 1 (Fig. S1).

### Demographic history

The estimate of mutation-scaled effective population size Θ was 7.6846, which converts to a historical (pre-whaling) effective population size of approximately 4000 breeding individuals. Estimates of contemporary (present time) N_e_ using an approximate Bayesian approach were 62 individuals with 95% confidence limits of 52.41–105.08 individuals.

The bottleneck analysis did not provide any evidence of a recent population decrease based on Wilcoxon test TPM model (*P* = 0.6875), as the expected heterozygosity (H_e_) at mutation-drift equilibrium was higher than the observed heterozygosity (H_o_) under a constant population size. Also, the qualitative descriptor of the allele frequency distribution was clearly L shaped, which is expected for non-bottleneck populations close to mutation-drift equilibrium (Fig. S2). In addition, the analyses of the *M-ratio* provided little support for a past bottleneck. Under both scenarios (Θ = 3 and Θ = 1; Ne of 1500 and 500 individuals), the observed *M-ratios* were larger (0.8883 and 0.9274) or not different from the calculated *Mc*. Moreover, both *M-ratios* were over the suggested threshold of 0.68 identified by Garza and Williamson (2001) for bottlenecked populations.

### Mitochondrial DNA diversity

Among the 46 sequenced individuals, a total of 12 different haplotypes were identified based on 12 polymorphic sites, 10 of which were parsimony informative. Of the 12 haplotypes detected, eight are new haplotypes for the blue whale (CH01, CH02, CH03, CH04, CH05, CH06, CH07, CH08; GenBank accession numbers JX035887 – JX035890 and KC116222 – KC116225). Because of the lack of polymorphic sites in the last 60 bp of the control region sequenced, the sequences were edited down to 360 bp for comparative analyses with previous reported sequences. After that, haplotypes CH05, CH06, CH07, and CH08 collapsed to match haplotypes P, S, O, and T reported by LeDuc et al. (2007). The other four haplotypes (different from CH01 to CH04) found in this study, matched those previously reported for the Pacific and/or the Antarctic Oceans (haplotypes Q, R, LL, DD) (LeDuc et al. 2007).

**Figure 3:**
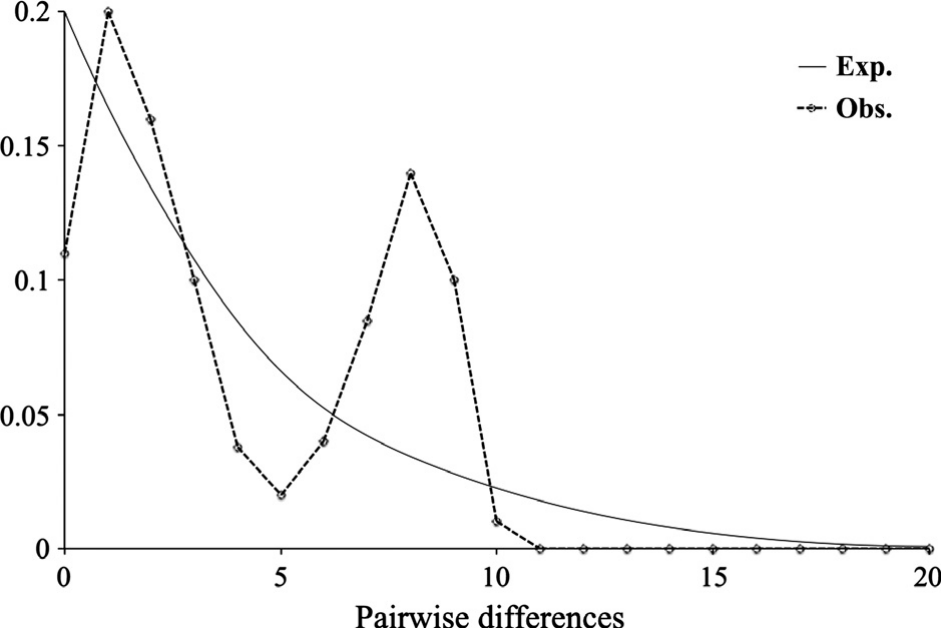
Mismatch distribution graph of the observed and expected mutations based on pairwise differences among haplotypes.

The mean number of pairwise differences (k) among the Corcovado Gulf blue whale haplotypes was 3.973 (± 0.79). Haplotype (Hd) and nucleotide (*π*) diversities resulted in Hd = 0.890 (± 0.019) and *π *= 0.011 (± 0.001), respectively (Table 2). No evidence of population expansion was found under a Fisher–Wright neutrality model, based on Fu's *F*s statistic (−0.657; *P *=* *0.435) and R2 test (0.1628; *P *=* *0.914). In addition, a mismatch distribution graph showed a bimodal distribution (Fig. 3), thus indicating no population expansion. A haplotype network was constructed for the Corcovado Gulf blue whales haplotype Q as the most frequent (19.6%) among the 12 detected haplotypes. The network did not reveal any central haplotype or star-like form (Fig. S3).

**Table 2:** Genetic diversity indexes in blue whales populations obtained using mtDNA for 4 known Southern Hemisphere feeding grounds (present study; LeDuc et al. 2007; Attard et al. 2010; Sremba et al. 2012).

Population	NH	Hd	*π*	GGD
Corcovado	12	0.890 (0.019)	0.011 (0.001)	0.26
Antarctica	26	0.968 (0.004)*	0.016 (0.0086)	0.55
Australia				
Bonney Upwelling	9	0.758 (0.070)*	0.004 (0.003)*	0.28
Perth Canyon	14	0.683 (0.062)*	0.003 (0.002)*	0.21

NH, Number of haplotypes; Hd, Haplotype diversity; *π*, nucleotide diversity; GGD, Gross Genetic Diversity Index.

* significant differences when compared to the Corcovado Gulf population.

### Genetic diversity among different feeding grounds and phylogenetic comparisons

Haplotype and nucleotide diversities found in the Corcovado Gulf were compared with those reported for other blue whale feeding areas (one in Antarctica and two in Australia). Haplotype diversity in Antarctica (Hd = 0.968) was higher than the Corcovado Gulf (Hd = 0.890; *P *<* *0.05), while nucleotide diversities (*π*) were not statistically different between both areas (Antarctica = 0.016; Corcovado Gulf = 0.011, *P *>* *0.05). When the Corcovado Gulf blue whale population was compared to both Australian feeding grounds, the Corcovado blue whale population was more diverse than the Perth Canyon and Bonney Upwelling for both haplotype and nucleotide diversities (*P *<* *0.05) (Perth Canyon [Hd = 0.683 *π *= 0.003] and Bonney Upwelling [Hd = 0.758 *π *= 0.004]) (Table 2) (LeDuc et al. 2007; Attard et al. 2010; Sremba et al. 2012). The gross genetic diversity index (GGD) was similarly lower for the Corcovado Gulf and both Australian populations, with the Antarctica population being the most genetically diverse (Table 2).

**Figure 4:**
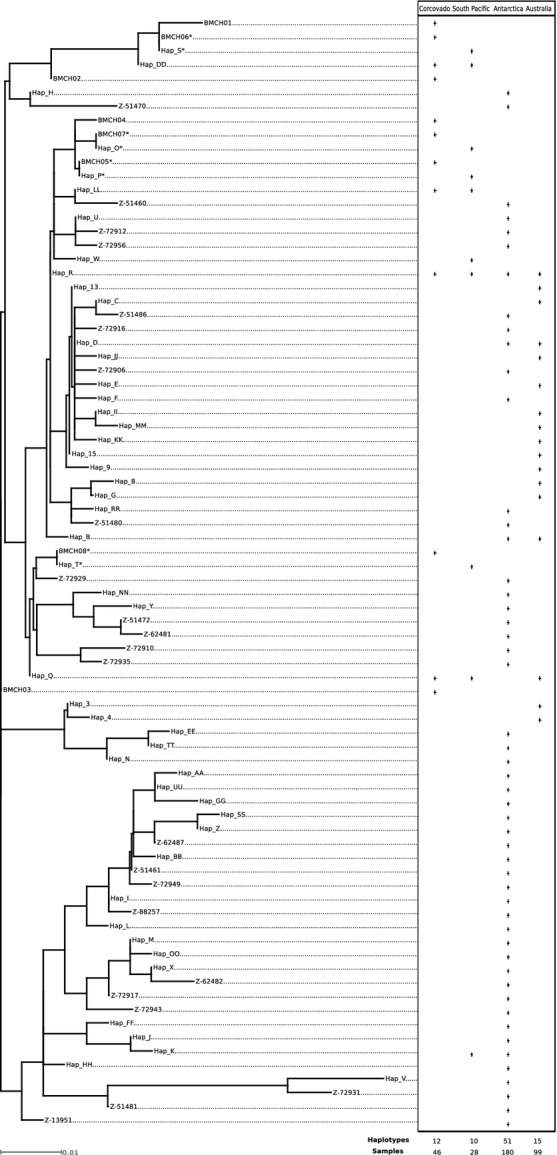
Maximum likelihood tree. Corcovado Gulf haplotypes (this study), Southeastern Pacific (LeDuc et al. 2007), Antarctica haplotypes (LeDuc et al. 2007; Sremba et al. 2012), Australia haplotypes (Attard et al. 2010).

Haplotype Q was the only haplotype to be shared between the Corcovado Gulf and Australian populations (Bonney Upwelling and Perth Canyon areas), while haplotype R was shared among all the three areas compared (Antarctica, Australia, and the Corcovado Gulf). The phylogenetic reconstruction of mtDNA haplotypes did not reveal complete lineage sorting among the three geographic areas, because only a few haplotypes were shared among populations. Therefore, no evidence for a possible subspecies differentiation was observed, as reported by other authors (LeDuc et al. 2007; Sremba et al. 2012) (Fig. 4).

## Discussion

### Genetic diversity and geographic differences

Reductions in population size are expected to lead to a loss of genetic diversity, which may affect the ability of populations to cope with heterogeneous environments and thus negatively influence species viability (Frankham 2005; Willi et al. 2006). However, despite this expectation, the results for the small blue whale population in the Corcovado Gulf did not show evidence of low levels of genetic diversity for either mitochondrial (e.g., haplotype diversity) or nuclear (e.g., number of alleles and heterozygosity) markers. This was unexpected, as this is not the general trend observed in several empirical studies of endangered mammal species, in which the most common situation reported is a significant strong decline of genetic diversity after populations have suffered from a bottleneck or in isolated and long-term hunted species (e.g., Taylor et al. 1994; Houlden et al. 1996; O'Ryan et al. 1998; Le Page et al. 2000; Hunter et al. 2010). However, it is worth noting that only a few studies have directly detected losses in genetic diversity for whale populations in the post-whaling era (e.g. Alter et al. 2012).

The unexpected high genetic diversity and lack of inbreeding (*F*_is_ = 0.023; *P *>* *0.05) observed in among the Corcovado Gulf blue whales could be explained by several different factors known to affect populations. A first hypothesis is that balancing selection could be favoring the retention of genetic variants in small and isolated populations; however, there is no evidence of isolation, so this explanation is hard to prove. Moreover, no sign of selection on mtDNA was found, as all alleles were neutral (Tajima's *D*,*P *>* *0.05). A second hypothesis could be that Corcovado Gulf blue whales belong to different populations that congregate in this single area to feed during the summer; however, the Bayesian clustering analysis did not show the Corcovado Gulf population as an admixture area. A third hypothesis is that not enough time has been elapsed since the end of the whaling (∼45 years); thus considering a blue whale population crash, the effects on heterozygosity will stay for 0.2–4 * N_e_ generations before a new equilibrium is set (Luikart and Cornuet 1998). Therefore, with a Ne of 62 individuals, it is then possible that a new equilibrium (He = Heq) has been set at 12.37 generations. A fourth hypothesis is that this population could be part of a larger population that segregates into several subpopulations during the feeding season as seen in other whales (e.g., humpback whales; Stevick et al. 2003); therefore, the genetic variability is expected to be higher than for small populations. This latter explanation in addition with the previous one seems the most plausible taking into account the genetic analyses of samples from other Southeastern Pacific (SEP) waters and the comparison performed on the data reported by LeDuc et al. (2007), thus suggesting the presence of a single population along the SEP (Torres 2011).

Alternatively but not exclusively, the Corcovado Gulf aggregation site could be a remnant population from the time of intensive whaling along the Chilean coast. Williams et al. (2011) calculated a population size of some thousands (2000–6200) blue whales before whaling in Chilean waters, of which at least 4288 were caught. Today, the estimated size of Chilean blue whale population range between 176 and 625 individuals, suggesting that whaling depleted this population to a minimum of 9.5% of its original size (Williams et al. 2011).

### Past and present effective population sizes and whaling bottleneck

Considering the historical pre-whaling and the present post-whaling effective population sizes N_e_, the decline in the number of individuals able to reproduce after the whaling harvest was high and significant. Thus, if the blue whales in the Corcovado Gulf were part of unique population along the Chilean coast, they are likely to have suffered a serious population bottleneck. However, we found no evidence of such event by any of the methods used in this research. We postulate that our data are not adequate to detect a genetic bottleneck event due to the relatively short time since the end of whaling (i.e., not enough generations to observe a loss of genetic diversity by drift). Indeed, the cease of blue whale catches in 1966, and the longevity and long generation time of an average blue whale (estimated at 70–100 years and ca. 31 years, respectively [Taylor et al. 2007]) account for just a few overlapped generations. This means some of the whales sampled in this study were still part of the original population, thus explaining the relatively higher genetic diversity and the lack of signs of a bottleneck event detected in this study (Amos 1996). Moreover, if Corcovado Gulf blue whales represent 9.5% of the previous population size, we would not expect a strong decline in diversity or a strong signal of a bottleneck.

Based on Willians et al. (2011) prewhaling population size range of 2000–6000 individuals for Chilean blue whales, the size of the Corcovado Gulf population may have been dramatically reduced to about 4–18% of its prewhaling size. Although the ratio N_total_/N_e_ is difficult to calculate in natural populations, theory suggests a ratio of 2 (Nunney and Elam 1994). Thus, correcting our past and present effective population sizes estimated in our study (3842 and 62 individuals, respectively) by a factor of 2, the results are at the same order of magnitude as the total population sizes estimated by Williams et al. (2011) for pre-whaling and post-whaling time.

### Comparison of the genetic diversity of blue whales in the Corcovado Gulf in relation to populations of other whale species

Although only a few published population genetics studies of blue whales are available, the data provided by LeDuc et al. (2007), Attard et al. (2010), and Sremba et al. (2012) are very useful for comparative purposes. This comparison showed that the blue whale population in the Corcovado Gulf has a lower genetic diversity than the Antarctica feeding grounds both at nuclear and mitochondrial markers, although a higher genetic diversity than the Australian feeding grounds at mitochondrial markers (because no nuclear comparison was possible due to different microsatellite loci used). The higher diversity in Antarctica can be explained by a sampling effect as the sampled individuals probably belong to different breeding grounds (i.e., populations) due to the fact that they were biopsied along the waters of an entire continent (i.e., circumpolar IWC management Areas). Because the identity of Chilean blue whales is unknown with respect to the other two populations (Australia and Antarctica), it cannot be assumed that Antarctica blue whale genetic diversity is representative of pre-exploitation genetic diversity in the Corcovado Gulf blue whales. In that sense, our study can be useful in terms of resolving some questions regarding the genetic structuring of blue whale populations.

The overall genetic diversity both at nuclear and mitochondrial markers among the Corcovado Gulf blue whales was similar to those reported for other baleen whales (e.g., H_o_: 0.63–0.82 and Hd: 0.67–0.91), including humpback (Engel et al. 2008; Cypriano-Souza et al. 2010), fin (Bérubé et al. 1998), sei (Kanda et al. 2006), brydes (Kanda et al. 2007), and minke whales (Pastene et al. 2010). All species except but one – the minke whale – were intensively captured during a relatively short period of time (19th–20th centuries). Other species hunted for a longer time, such as the North Atlantic right whale (11th–20th centuries) appeared to lose a greater amount of genetic diversity (H_o_ = 0.31–0.42; Waldick et al. 2002), or have shown that even where diversity remains high, maternal lineages were likely lost from prewhaling populations (e.g. Alter et al. 2012). Thus, our data support the hypothesis that not enough time has elapsed since the cessation of whaling to show a clear effect of whaling on the genetic diversity in the Corcovado Gulf blue whales.

### Relationship among different feeding grounds

Based on the number of shared haplotypes, very little overlap between the Corcovado Gulf, Antarctica and Australia blue whales populations was found. Moreover, most haplotypes were found to be private to a single area, suggesting isolation between populations in these three feeding grounds. Although the phylogenetic tree shows some geographical separation between the mtDNA lineages sampled among three feeding grounds, some haplotypes seem to be more similar to haplotypes of other feeding grounds than those belonging to the same feeding ground. This could be due to a recent common ancestor rather than to an exchange of individuals between areas, thus resulting in an incomplete lineage sorting. Comprehensive analyses using both mitochondrial and nuclear markers should be carried out in order to estimate the existence and magnitude of gene flow and their effects on population genetic differentiation, including exchange with blue whales from other areas such as the Eastern Tropical Pacific and the Indian Ocean.

Finally, studies on phylogenetic relationships with other blue whale feeding grounds along the Chilean coast will be needed to assess if the blue whale population in the Corcovado Gulf area is part of a larger population. Additionally, the degree of connectivity among blue whale populations within the Pacific basin should be assessed to achieve a clearer view of putative breeding areas (all of which are unknown), as well as to define the most appropriate management units (*sensu* Donovan 1991).

### Sampling and laboratory considerations

It should be noted that our work is based on a small number of samples that may have reduced the statistical power of the analysis. Nonetheless, this problem is most pronounced when samples are small in relation to the actual population size (Belkhir et al. 2006). In this case, this is unlikely because the population size of blue whales in Corcovado Gulf area is known to be small (Williams et al. 2010; Galletti Vernazzani et al. 2012). Also, it is unlikely that increasing the sample size would increase the accuracy of the genetic diversity estimates, as a sample size ranging between 25 to 30 diploid individuals should be sufficient to obtain significant results (Hale et al. 2012). It is expected that the use of different molecular markers (nDNA and mtDNA) would provide different results because they evolve at different rates and have different modes of inheritance (Ballard and Whitlock 2004). Although contrary to what is expected for microsatellites markers, mitochondrial genetic diversity does not necessarily have a straightforward relationship with population size (Bazin et al. 2006). Despite the previous statement, the number of individuals sampled, the small population size, and the number of loci (nuclear and mitochondrial) used in our study indicate that other conservation studies can accomplish the same questions we explored here, particularly when sample sizes are small.

### Conservation implications

Although the high genetic diversity and the absence of any significant genetic bottleneck during the last century in the Corcovado Gulf blue whale feeding ground suggest that this population has a good chance of long-term viability (Frankham 2005), this conclusion must be taken with care, however, as there are no signs of population expansion and the abundance of blue whales in the area is still considered to be low. Additionally, although national and international organizations have established principles and strategies for monitoring biological diversity (i.e., IUCN, Convention on Biological Diversity; McNeely et al. 1990), the information provided by molecular markers still could be more effectively integrated into those strategies. Genetic data should not only be used to study demography or more complex evolutionary and ecological processes, but to estimate population genetic parameters over time and not just as single “snapshots” (Schwartz et al. 2007). Our data represent the foundation or current baseline for future population genetic assessments of Chilean blue whales for which comparisons can be made as recovery from whaling continues.

We recommend a systematic genetic monitoring program for blue whales on this feeding ground (*sensu* Laikre et al. 2009). Furthermore, any monitoring program should be combined with demographic studies, evaluating environmental variables and human impacts on populations (Frankham 2010), including local measures to protect the habitat of specific local populations (see Hucke-Gaete et al. 2006).

Finally, we hope this study can contribute to the establishment of a multiple-use marine-protected area in the region (Hucke-Gaete et al. 2010), which would aid in the recovery of the blue whale of the Corcovado Gulf feeding ground and consequently the long-term monitoring of this species.

## Supporting Information

**Figure S1.** Bayesian analysis graph, showing no structure among Corcovado Gulf blue whales population. **Figure S2.** Allele frequency distribution in the blue whale Corcovado feeding ground. **Figure S3.** Neighbor-joining network showing the relationships among Corcovado Gulf blue whale haplotypes.
