# Supplementary material for: High genetic diversity in a small population: the case of Chilean blue whales
Source: Ecol Evol. 2014 Mar 20;4(8):1398–412. doi: 10.1002/ece3.998 (PMC4020699; doi:10.1002/ece3.998)
Supplement: Supplementary file 1 — Figure S1. Bayesian analysis graph, showing no structure among Corcovado Gulf blue whales population. Figure S2. Allele frequency distribution in the blue whale Corcovado feeding ground. Figure S3. Neighbor-joining network showing the relationships among Corcovado Gulf blue whale haplotypes. [file ece30004-1398-sd1.doc]

APPENDIX

**Figure 1**. Bayesian analysis graph, showing no structure among Corcovado Gulf blue whales population.

**Figure 2**. Allele frequency distribution in the blue whale Corcovado feeding ground. The bars represent the percentage of all alleles detected in each allele frequency class, showing an L distribution as a visual sign of no recent bottleneck


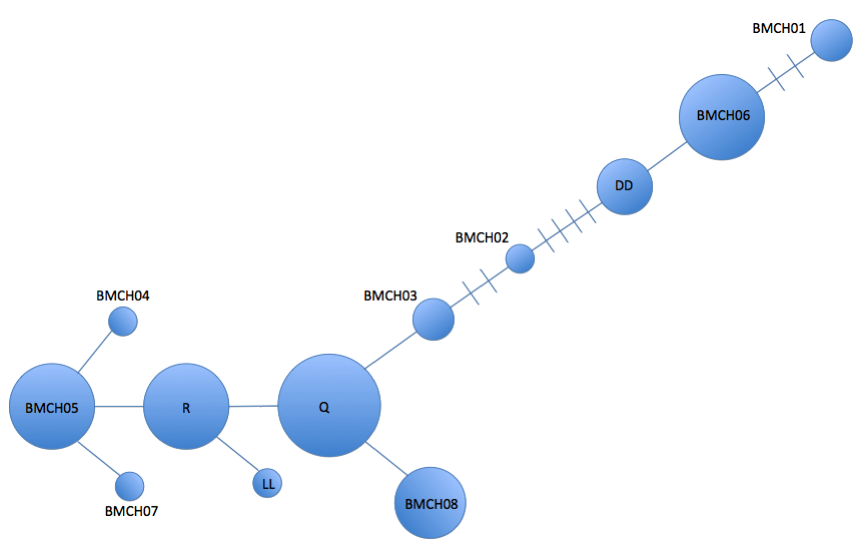


**Figure 3.** Neighbor-joining network showing the relationships among Corcovado Gulf blue whale haplotypes. Each circle represents one haplotype and sizes indicate the frequency of each haplotype. Each overcrossing line represents one base pair change between sequences. Codes BMCH are unique haplotypes for Corcovado Gulf region, Q, R, LL, DD are haplotypes reported previously in other blue whale populations (LeDuc et al. 2007).
